# Flourishing and job satisfaction in employees working in UK clinical trial units: a national cross-sectional survey

**DOI:** 10.1186/s12913-024-11986-x

**Published:** 2024-12-02

**Authors:** Sophie S. Hall, Evgenia Riga, Kirsty Sprange, Pamela Hagan, Lucy Carr, Jodi Taylor, Louise Thomson, Eleanor J. Mitchell

**Affiliations:** 1 Nottingham Clinical Trials Unit, University of Nottingham, Nottingham, UK; 2 Faculty of Medicine and Health Sciences, University of Nottingham, Nottingham, UK; 3 Sheffield Clinical Trials Research Unit, University of Sheffield, Sheffield, UK; 4 Medical School, University of Bristol, Bristol, UK

**Keywords:** Clinical trials, Research staff, Flourishing, Workplace wellbeing, Job satisfaction

## Abstract

**Background:**

To evaluate healthcare interventions in clinical trials, it is crucial to attract and retain a skilled workforce. The job demands associated with developing and running clinical trials have been linked with difficulties in recruiting and retaining skilled Clinical Trial Unit (CTU) staff. Flourishing conceptualises positive aspects of wellbeing which may help staff to thrive within a demanding job role. This study explored the association between flourishing and job satisfaction among staff based in UK Clinical Research Collaboration (UKCRC)-registered CTUs.

**Methods:**

A national online survey of UKCRC-registered CTUs was conducted which combined psychometric measures of flourishing (eudaimonic workplace wellbeing scale) and job satisfaction (including measures of turnover intention and workplace engagement), alongside free-text questions.

**Results:**

Four hundred and eighty-four staff from 52 UKCRC CTUs completed the survey. Overall, participants reported 'average’ levels of job satisfaction and work engagement, but there was evidence that CTU staff reported slightly lower levels of flourishing and moderate levels of turnover intention. Salary, role, and flexible working arrangements were associated with levels of flourishing. When these factors were controlled for, higher levels of flourishing were still predictive of job satisfaction and turnover, but not work engagement. Qualitative analysis of free text responses revealed that elements of the working environment, such as supportive relationships, flexible working, and development opportunities, can act as resources to help employees flourish in their jobs.

**Conclusions:**

Through exploring flourishing in CTU staff we identified factors which may help CTU employees thrive in their role, and in turn increase job satisfaction and commitment to their place of work. CTUs should consider the importance of developing a working environment which supports staff to feel valued, experience autonomy and supportive working relationships, as well as opportunities to develop and engage in meaningful work. Efforts to understand and protect the wellbeing of CTU staff are vital to attract and retain staff to design and conduct clinical trials. The learning from this may be applicable to other healthcare workforces facing a recruitment and retention crisis.

**Supplementary Information:**

The online version contains supplementary material available at 10.1186/s12913-024-11986-x.

## Background

Staff working in Clinical Trial Units (CTU) play a pivotal role in shaping and executing high-quality healthcare research [1, 2]. A priority for the future of clinical research is to recruit and retain expert staff [3–8]. Building and sustaining talent within the research and development workforce is a strategic priority to help protect and improve the health of the nation [9].

In the UK, clinical trials are typically undertaken in collaboration with CTUs, who employ staff with expertise in designing, coordinating, analysing, and reporting clinical trials. The efficiency of CTUs is paramount in delivering timely responses to healthcare priorities; however, clinical trials can often be inefficient [10–13]. Reports highlight common issues such as inadequate study team management, lack of motivation and experience among trial team members, as well as insufficient staffing [8, 13–15]. Notwithstanding the limited published evidence on the global turnover rates of clinical trial staff, a report from a U.S. industry-funded clinical research study revealed that in 2021, 32% of trial associates left their positions [16]. Furthermore, there is widespread international concern CTUs are facing challenges in attracting and retaining qualified personnel [4, 17–19].

The reasons for these recruitment and retention challenges are not well documented. However, it is acknowledged that working in a CTU is associated with specific work demands, including administrative burden, and the ability to work cohesively within a multi-disciplinary team (e.g., statisticians, data managers, clinical trialists, quality assurance teams, administrators) [17, 19]. Furthermore, operating within a dynamic, time-sensitive context, CTU staff must exhibit adaptability to accommodate recent scientific advancements, shifting priorities, and societal changes [4, 17]. Ultimately, these challenges could lead to stress, burnout, and a staffing shortage [17, 20] which further exacerbates job demands on the remaining staff.

One way of exploring recruitment and retention issues is through exploring workplace wellbeing. Workplace wellbeing is often discussed through concepts such as stress and anxiety and psychosocial factors, such as job demands, having a negative impact on employee wellbeing and organisational commitment [21–23]. This approach captures specific (often negative) emotions within a short timeframe (e.g., anxiety over the last week) and is congruent with the concept of ‘hedonic’ wellbeing [24]. In contrast, eudaimonic wellbeing, also called ‘flourishing’ conceptualises positive aspects of wellbeing, including self-acceptance, positive relations with others, autonomy, environmental control, purpose in life and personal growth [25]. This approach encompasses individual development through engagement with life challenges, being resilient, and being able to fully function [24–26]. Recently, attempts have been made to evaluate flourishing in a workplace context. This has led to the development of the two-dimensional Eudaimonic Workplace Wellbeing Scale (EWWS), which conceptualises flourishing through two factors, interpersonal and intrapersonal flourishing [27].

Interpersonal flourishing focuses on external and social factors that shape the individual’s working experience. It draws upon concepts such as social support and acceptance leading to a sense of ‘belonging’ in the workplace [27]. Evidence across different professions highlights the importance of interpersonal factors in shaping job satisfaction [28–30], organisational commitment, and turnover intention [31, 32]. Social support is proposed to buffer against role demands, reducing stress and feelings of isolation, while fostering sharing of coping strategies and therefore growth [30, 33].

Intrapersonal flourishing focuses on the internal and personal factors that influence the working experience. It encompasses both value and meaning obtained from the working environment, as well as the concepts of autonomy (ability to make decisions/determine direction) and environmental mastery (ability to control one’s environment) [27]. Control over the work environment, such as flexible working arrangements, are thought to promote a sense of indebtedness in employees such that it enhances organisational commitment and decreases turnover [34–36].

In summary, workplace flourishing represents a positive state of wellbeing that can be influenced by the presence of job resources and the effective management of job demands [37], aligning with established models of occupational stress, such as the Job Demands-Resources (JD-R) model [38, 39]. The JD-R model is a framework used to understand employee well-being and performance by balancing two key factors: job demands and job resources. Job Demands are the aspects of a job that require sustained effort (e.g., workload, emotional strain), these demands can lead to stress and burnout. Job Resources are the factors that help employees meet, or buffer against, job demands (e.g., support from colleagues, autonomy, career opportunities). Flourishing may occur when job resources are sufficient to offset job demands, enhancing engagement, satisfaction and commitment. In contrast, languishing may occur if job demands outweigh job resources, leading to low job satisfaction. Therefore, we hypothesised that higher levels of flourishing would be associated with higher levels of work engagement and job satisfaction and lower levels of turnover intention. We also hypothesised that levels of flourishing may be associated with specific workplace characteristics, and identifying these would be important for identifying ‘at-risk’ employees.

While the role of flourishing among CTU staff has not been directly investigated, the unique work demands faced by these employees—including the ability to effectively collaborate within a diverse team, adapt to evolving scientific priorities, navigate a constantly changing regulatory environment, and maintain sustained effort over time [40]—suggest that understanding flourishing could be a valuable pathway to enhancing job satisfaction and commitment among CTU staff.

## Methods

### Aims and Objectives

The aim of this study was to gain insight into the relationship between flourishing and job satisfaction among CTU staff, by addressing four objectives. Throughout we use the term job satisfaction to encompass measures of turnover intention and workplace engagement as well as job satisfaction per se. Specific objectives were to:

- Identify current levels of flourishing and job satisfaction in UK CTU staff.
- Identify workplace characteristics associated with individuals scoring high and low on workplace flourishing measures to facilitate the identification of employees at risk of not flourishing in CTUs.
- Explore the relationship between workplace flourishing and measures of job satisfaction to identify if flourishing is associated with retaining satisfied CTU staff.
- Explore pathways to flourishing within a CTU workplace.

### Design and Setting

Cross-sectional survey of UKCRC-registered CTU employees.

### Ethical Approval

The study received ethical approval from the University of Nottingham Faculty of Medicine and Health Sciences Research Ethics Committee (FMHS 101–1022). Participants provided consent online before completing the survey.

### Participants

Participants were recruited via advertisements on social media (e.g., X formally known as Twitter) and invitations to all UKCRC-registered CTU Directors or named representatives for local distribution within their CTU. Study advertisements were shared through established networks (including the UK Trial Managers' Network (UKTMN) and the Trials and Methodology Research Partnership (TMRP) working groups). Participants were required to confirm that they worked in a UKCRC-registered CTU for at least three months and be currently employed by a UKCRC-registered CTU, before providing consent to participate. In collaboration with the Study Advisory Group, a 3-month minimum employment period was chosen. This was to ensure participants had some experience with their role and CTU, and to avoid only recruiting employees who were well established in their CTU, which may provide a bias sample of employees with low turnover intention. The survey was open to all CTU employees, irrespective of role, to promote representation across the full spectrum of positions.

### Instruments

Please see Supplementary Material 1 for the survey items, developed for the purpose of this study.

#### Demographic survey

Items assessed the defining characteristics of the participant (gender, ethnicity, age) and defining characteristics of their place of employment (length of time at employment, type of contract, salary scale, CTU’s hybrid working policy, main job role, flexible working policy arranged with line manager). In addition, we also asked participants to state their preferred hybrid working arrangement.

#### Eudaimonic Workplace Wellbeing Scale (EWWS)

Assesses interpersonal and intrapersonal flourishing in the workplace [27]. The EWWS uses a 5-point scale (1 = strongly disagree, 5 = strongly agree) to assess interpersonal (4 items) and intrapersonal (4 items) flourishing. Higher scores suggest greater flourishing in the workplace.

#### Utrecht Work Engagement Scale (UWES)

A three-factor model measuring work engagement through nine items relating to employee vigour, dedication and absorption; as well as total work engagement [37, 41]. Each item is scored using a 7-point scale (never = 0, always/every day = 6). Higher scores are indicative of greater work engagement.

#### Job Satisfaction Scale (JSS)

A unidimensional scale assessing employee job satisfaction [42] through 10 items rated on a 5-point scale (Strongly disagree = 1; Strongly agree = 5). Higher scores are indicative of higher job satisfaction.

#### Turnover Intention Scale (TIS)

A unidimensional scale measuring an employee’s intention to leave their current place of employment [43, 44] through 6-items, rated on a 5- point scale (1 = never, 5 = always). One item is reverse scored. Higher scores are indicative of greater intention to leave the participant’s current place of employment.

#### Open-ended Response Items

Compulsory open-ended questions were included at the end of the survey asking for details on what made individuals feel satisfied/dissatisfied at work.

- What is it about your working environment (e.g., things you do, or things your CTU does) that makes you feel satisfied at work?
- What is it about your working environment (e.g., things you do, or things your CTU does) that makes you feel dissatisfied at work?

### Data Collection

Data were collected using the Jisc Online Survey tool, and after user-testing by 10 individuals, the survey was open between January 13th and February 28th 2023.

### Data Analysis

Data were analysed using IMB SPSS 28 software. Since this research is the first large-scale investigation into flourishing and job satisfaction among staff working in UK CTUs, our initial aim was to describe their current levels, providing a baseline for future research to enable comparisons over time. Descriptive statistics (Mean ± Standard Error Mean) were computed and compared against their manuals cut-off points. Where no formal scoring manual or interpretation was available (EWWS and TIS) data was interpreted through comparison of our sample’s scores with data from other research in the workforce.

The second part of describing current levels of flourishing and job satisfaction was to explore whether flourishing and job satisfaction levels are the same or different across key workplace characteristics. This is important to help us identify potentially ‘at-risk’ employees. Therefore, Analysis of Variance (ANOVAs) were conducted. The workplace characteristics associated with significant differences in flourishing and job satisfaction were subsequently controlled for in two-step regression models. The purpose of the regression models was to explore whether levels of workplace flourishing (interpersonal and intrapersonal) were associated with measures of job satisfaction, even when controlling for key workplace characteristics (identified in the aforementioned analysis). This is important to help CTU directors and operational managers evaluate whether incorporating flourishing in their wellbeing policies is likely to bring a positive impact to their units staffing.

Finally, we sought to explore the pathways which may lead to high levels of flourishing so that we can begin to develop models and recommendations to support CTU flourish in their work. To this end, we selected the highest and lowest scoring participants on the intrapersonal and interpersonal scales of flourishing until we reached a minimum sample size of 82 for each group. The sample size was determined through a power calculation to ensure sufficient numbers were included to detect a moderate practical difference between the two groups. Where multiple participants achieved the designated cut-off score for inclusion in a group all were included in their respective group. Selecting these groups (high and low flourishers) enabled us to feasibly conduct a more in-depth qualitative evaluation from a large data set. The free-text responses provided by the high and low flourishers to the questions ‘What makes you feel satisfied at work?’ and ‘What makes you feel dissatisfied at work?’, were analysed using a mixture of deductive and inductive approaches. This enabled key narratives to emerge from the data [45]. We first adopted a deductive approach to broadly code the data using key concepts included in a flourishing framework, including interpersonal elements (e.g., social support, sense of belonging) and intrapersonal elements (e.g., autonomy, growth, purpose). We then used an inductive approach to explore for themes within these codes. Throughout the analysis a constructivist epistemology was taken by the two researchers who independently coded the data, with decisions reviewed and disagreements resolved through discussions. A constructivist approach was taken to enable us to explore how peoples working environment and experiences contribute to their levels of flourishing and job satisfaction.

## Results

### Participant characteristics

Four hundred and eighty-four CTU staff participated, with representation from all 52 UKCRC-registered CTUs (at time of data collection) (Table 1). In summary, most participants identified as White, female, and aged 26–46 years.

**Table 1:** Participant Characteristics (*n* = 484)

	**n (%)**	**Job Satisfaction**	**Turnover Intention**	**Overall Work Engagement**	**Vigour**	**Dedication**	**Absorption**	**Total Flourishing**	**Interpersonal Flourishing**	**Intrapersonal Flourishing**
**Total Sample**	484 (100%)	35.73 ± .28	2.57 ± 0.04	3.88 ± .04	3.33 ± .05	4.27 ± .04	4.06 ± .04	29.00 ± .24	14.22 ± .14	14.77 ± .13
***Gender***										
Male	97 (20%)	35.67 ± 0.68	2.58 ± 0.11	3.87 ± .10	3.34 ± 0.12	4.27 ± 0.10	3.99 ± 0.10	28.46 ± 0.56	13.94 ± 0.32	14.51 ± 0.30
Female	376 (78%)	35.87 ± 0.31	2.55 ± 0.05	3.88 ± .04	3.32 ± 0.61	4.25 ± 0.57	4.07 ± 0.04	29.20 ± 0.27	14.31 ± 0.16	14.88 ± 0.14
Non-binary	3 (< 1%)	31.33 ± 1.45	2.77 ± 0.54	4.07 ± .16	3.44 ± 0.22	4.55 ± 0.22	4.22 ± 0.48	29.33 ± 3.17	14.33 ± 1.45	15.00 ± 1.73
Prefer not to say	8 (2%)	31.75 ± 2.24	3.14 ± 0.23	4.15 ± .22	3.50 ± 0.40	4.66 ± 0.14	4.29 ± 0.19	26.00 ± 2.05	13.25 ± 1.53	12.75 ± 0.77
***Age***										
18–25 years	33 (7%)	35.21 ± 1.07	2.38 ± 0.17	3.97 ± .16	3.50 ± 0.22	4.31 ± 0.19	4.10 ± 0.14	28.87 ± 0.93	13.78 ± 0.61	15.09 ± 0.44
26–36 years	173 (36%)	35.58 ± 0.43	2.64 ± 0.07	3.96 ± .07	3.40 ± 0.08	4.35 ± 0.07	4.13 ± 0.07	28.77 ± 0.40	14.08 ± 0.25	14.69 ± 0.20
37–46 years	149 (31%)	35.72 ± 0.48	2.61 ± 0.08	3.84 ± .07	3.26 ± 0.09	4.21 ± 0.08	4.05 ± 0.07	29.08 ± 0.43	14.14 ± 0.26	14.93 ± 0.23
47–56 years	95 (19%)	35.76 ± 0.70	2.52 ± 0.12	3.74 ± .11	3.16 ± 0.14	4.15 ± 0.13	3.90 ± 0.10	29.02 ± 0.61	14.55 ± 0.32	14.46 ± 0.35
57–66 years	24 (5%)	38.08 ± 1.60	2.22 ± 0.24	3.99 ± .19	3.55 ± 0.22	4.29 ± 0.19	4.12 ± 0.21	30.41 ± 1.00	14.95 ± 0.73	15.45 ± 0.58
> 66 years	0 (0%)									
Prefer not to say	10 (2%)	34.20 ± 1.53	2.80 ± 0.21	4.13 ± .17	3.50 ± 0.32	4.66 ± 0.11	4.23 ± 0.16	28.70 ± 1.52	14.60 ± 0.87	14.10 ± 0.87
***Ethnicity***										
White	428 (88%)	35.92 ± 0.29	2.55 ± 0.05	3.89 ± .04	3.34 ± 0.05	4.28 ± 0.05	4.06 ± 0.04	29.17 ± 0.25	14.32 ± 0.15	14.85 ± 0.13
Asian/Asian Brit	31 (6%)	33.96 ± 1.28	2.68 ± 0.19	3.57 ± .18	2.97 ± 0.22	3.84 ± 0.23	3.88 ± 0.16	27.38 ± 1.29	13.19 ± 0.69	14.19 ± 0.68
Black/Black-Brit, Carib., African	5 (1%)	36.40 ± 2.18	2.40 ± 0.37	3.84 ± .23	3.22 ± 0.20	4.26 ± 0.33	4.06 ± 0.24	26.60 ± 2.18	13.00 ± 1.81	13.60 ± 0.67
Mixed/multiple	6 (1%)	35.16 ± 3.60	2.94 ± 0.42	4.27 ± .54	3.94 ± 0.53	4.50 ± 0.60	4.38 ± 0.60	29.50 ± 2.96	14.83 ± 1.83	14.66 ± 1.3
Other	12 (3%)	33.66 ± 1.74	2.70 ± 0.23	4.31 ± .26	3.69 ± 0.39	4.75 ± 0.21	4.47 ± 0.24	27.66 ± 1.60	13.83 ± 0.99	13.83 ± 0.86
Prefer not to say	2 (< 1%)	35.00 ± 8.00	3.50 ± 0.16	3.55 ± .33	2.83 ± 0.16	3.83 ± 0.16	4.00 ± 0.66	29.00 ± 0.00	13.50 ± 0.50	15.50 ± 0.50
***Salary***										
Up to £30,999	90 (19%)	34.72 ± .67	16.10 ± .73	4.05 ± .09	3.55 ± .11	4.39 ± .10	4.21 ± .09	27.93 ± .67	13.88 ± .38	14.04 ± .37
£31,000-£40,999	176 (36%)	35.11 ± .45	15.78 ± .46	3.85 ± .07	3.30 ± .09	4.21 ± .08	4.03 ± .07	28.77 ± .38	14.16 ± .24	14.60 ± .20
£41,000-£50,999	137 (28%)	36.48 ± .52	15.24 ± .54	3.87 ± .08	3.28 ± .10	4.28 ± .09	4.04 ± .07	29.24 ± .45	14.31 ± .27	14.92 ± .24
> £51,000	71 (15%)	37.11 ± .66	14.56 ± .68	3.77 ± .10	3.19 ± .13	4.18 ± .11	3.94 ± .11	30.66 ± .55	14.80 ± .35	15.85 ± .27
Prefer not to say	10 (2%)	35.70 ± .2.59	13.00 ± .2.30	4.08 ± .32	3.30 ± .47	4.60 ± .31	4.36 ± .24	27.70 ± .1.92	13.10 ± .92	14.60 ± .1.14
***Role in CTU***										
Trial Management	225 (46%)	35.40 ± .37	16.20 ± .40	3.89 ± .06	3.35 ± .07	4.26 ± .07	4.05 ± .06	28.75 ± .34	14.14 ± .20	14.60 ± .18
Data & IT	79 (16%)	34.59 ± .70	15.97 ± .74	4.07 ± .10	3.52 ± .13	4.49 ± .11	4.19 ± .10	27.65 ± .62	13.88 ± .38	13.77 ± .32
Statistics and HE	76 (16%)	36.36 ± .79	13.96 ± .72	3.82 ± .11	3.21 ± .14	4.16 ± .13	4.09 ± .10	29.59 ± .66	14.22 ± .41	15.36 ± .34
Other	68 (14%)	36.52 ± .81	14.51 ± .85	3.81 ± .10	3.22 ± .12	4.21 ± .12	4.00 ± .10	29.76 ± .71	14.55 ± .41	15.16 ± .37
Senior Managers	36 (7%)	37.42 ± .96	14.58 ± .94	3.76 ± .18	3.22 ± .22	4.19 ± .20	3.87 ± .17	30.94 ± .74	14.88 ± .44	16.05 ± .40
***Years of Employment***										
3 months – 3 years	204 (42%)	35.78 ± .42	2.37 ± 0.12	3.85 ± .07	3.31 ± .08	4.22 ± .08	4.03 ± .06	29.15 ± .037	14.11 ± .22	15.03 ± .20
4–9 years	173 (36%)	35.68 ± .45	2.65 ± 0.08	3.95 ± .06	3.41 ± .08	4.37 ± .07	4.08 ± .06	28.91 ± .41	14.25 ± .25	14.65 ± .21
10 years plus	107 (22%)	35.72 ± .65	2.70 ± 0.10	3.84 ± .09	3.22 ± .11	4.19 ± .11	4.09 ± .09	28.86 ± .52	14.40 ± .30	14.46 ± .29
***Type of Contract***										
Fixed term	241 (50%)	35.44 ± .39	2.57 ± 0.06	3.89 ± .06	3.35 ± .07	4.25 ± .07	4.08 ± .06	29.09 ± .36	14.29 ± .21	14.80 ± .19
Permanent	228 (47%)	36.10 ± .41	2.55 ± 0.07	3.88 ± .06	3.30 ± .08	4.29 ± .07	4.04 ± .06	29.04 ± .33	14.20 ± .21	14.83 ± .18
Unsure/other	15 (3%)	34.80 ± 1.52	2.87 ± 0.27	3.88 ± .15	3.28 ± .30	4.24 ± .22	4.13 ± .14	26.86 ± 1.53	13.40 ± .91	13.46 ± .78
***CTU’s Hybrid/Working from Home (WfH) policy***										
100–60% office	79 (17%)	34.58 ± .74	2.70 ± 0.12	3.82 ± .09	3.26 ± .11	4.19 ± .10	4.00 ± .10	28.25 ± .65	13.83 ± .35	14.41 ± .36
40–20% office	275 (56%)	36.06 ± .37	2.51 ± 0.06	3.85 ± .06	3.27 ± .07	4.24 ± .06	4.05 ± .05	29.36 ± .32	14.47 ± .20	14.89 ± .17
Flexi/Ad-hoc	115 (24%)	36.10 ± .55	2.57 ± 0.10	4.01 ± .08	3.50 ± .10	4.39 ± .09	4.14 ± .08	28.86 ± .48	14.00 ± .29	14.86 ± .26
Don’t know	15 (3%)	32.93 ± 1.21	2.94 ± 0.24	3.86 ± .27	3.33 ± .32	4.24 ± .32	4.02 ± .24	27.40 ± .122	13.46 ± .88	13.93 ± .65
***Preferred WfH policy***										
100–60%	59 (15%)	35.54 ± 0.81	2.60 ± 0.13	3.95 ± .14	12.30 ± 0.47	15.42 ± 0.45	15.06 ± 0.37	29.01 ± 0.75	14.30 ± 0.47	14.71 ± 0.38
40–20%	217 (45%)	35.74 ± 0.43	2.53 ± 0.07	3.81 ± .06	12.99 ± 0.24	15.85 ± 0.23	15.12 ± 0.19	28.76 ± 0.37	14.02 ± 0.22	14.74 ± 0.19
Flexible	208 (43%)	35.78 ± 0.40	2.61 ± 0.07	5.78 ± .06	13.18 ± 0.24	15.88 ± 0.21	15.31 ± 0.19	29.24 ± 0.35	14.41 ± 0.21	14.82 ± 0.19
***Flexible Working Policy Agreement in Place with Line Manager***										
Yes	257 (53%)	36.41 ± .36	2.54 ± 0.06	3.84 ± .06	3.25 ± .07	4.21 ± .07	4.05 ± .05	29.56 ± .32	14.45 ± .19	15.10 ± .18
No	227 (47%)	34.96 ± .42	2.61 ± 0.06	3.94 ± .06	3.41 ± .07	4.33 ± .06	4.07 ± .06	28.37 ± .37	13.97 ± .22	14.40 ± .19

Workplace characteristics are detailed in Table 1. Most participants held the role of trial manager (46%). The distribution of employment contracts showed a roughly equal split between permanent/open-ended and fixed-term contracts. More than half (56%) of participants stated that their CTU mandated employees to be present in the office for two days or less per week, with ad-hoc office working as the second most common category selected (24%). Preferences among respondents were strongly weighted towards flexible office arrangements (43%) and working in the office for two days or less per week (45%). Notably, more than half of participants (53%) had a flexible working policy in place, as agreed upon with their line manager.

### Cross-sectional survey *bias*

Given the cross-sectional survey nature of the study we assessed for the presence of common method bias through Harman’s single-factor test using exploratory factor analysis (EFA). All variables relating to flourishing and job satisfaction were entered into the analysis, and a principal axis factoring method was employed, constraining the extraction to a single factor. According to Harman’s test, if common method bias is a concern, a single factor would account for the majority of the variance in the dataset. The results indicated that the first factor accounted for 27.68% of the total variance, which is below the recommended threshold of 50%. Therefore, common method bias does not appear to be a major concern. Correlations between the survey items are presented in Supplementary Material 2.

### Current levels of flourishing and job satisfaction in CTU staff

Full results can be found in Table 2 and are summarised below.

**Table 2:** Characteristics of participant place of employment and measures of flourishing and job satisfaction

	**Interpersonal Flourishing**			**Intrapersonal Flourishing**			**Job Satisfaction**			**Turnover Intention**			**Vigour**			**Dedication**			**Absorption**		
	*p* <	**CI 95%** **Lower**	**CI 95%** **Upper**	*p* <	**CI 95%** **Lower**	**CI 95%** **Upper**	*p* <	**CI 95%** **Lower**	**CI 95%** **Upper**	*p* <	**CI 95%** **Lower**	**CI 95%** **Upper**	*p* <	**CI 95%** **Lower**	**CI 95%** **Upper**	*p* <	**CI 95%** **Lower**	**CI 95%** **Upper**	*p* <	**CI 95%** **Lower**	**CI 95%** **Upper**
**CTU WFH Policy**	.76	.00	.03	.21	.00	.02	.07	.00	.04	.28	.00	.03	.35	.00	.02	.56	.00	.02	.75	.00	.01
**Role**	.76	.00	.02	.001	.01	.08	.09	.00	.04	.04	.00	.05	.43	.00	.02	.34	.00	.03	.53	.00	.02
**Salary**	.34	.00	.03	.02	.01	.07	.04	.00	.04	.35	.00	.03	.35	.00	.03	.56	.00	.02	.33	.00	.03
**Flexi Agreement**	.11	.00	.03	.01	.001	.04	.01	.001	.04	.49	.00	.01	.15	.00	.02	.25	.00	.02	.85	.00	.01
**Years at CTU**	.76	.00	.01	.21	.00	.03	.99	.00	.00	.06	.00	.04	.42	.00	.02	.29	.00	.02	.84	.00	.01
**Type of Contract**	.76	.00	.02	.21	.00	.03	.44	.00	.02	.52	.00	.02	.88	.00	.01	.92	.00	.01	.86	.00	.01

Where possible we compared mean scores against the criteria set out in the scales scoring manual. Where no scoring manual or criteria existed we compared our samples score to available published data.

Mean scores for interpersonal flourishing (14.22 ± 0.14; Mean ± SEM) and intrapersonal flourishing (14.77 ± 0.13), were compared to other samples using the same EWWS scale (see Supplementary material 3). This comparative review process suggested our sample scored slightly below that of other samples, indicating the possibility that CTU staff may experience lower levels of flourishing lower than those working in other organisations. It should be noted that other factors (e.g., comparisons across cultures and different sample sizes) could influence this interepreation.

The mean score on the measure of job satisfaction was 35.73 ± 0.28. In accordance with the scale’s scoring manual, this indicates that job satisfaction in CTU staff at the time of data collection was average [46].

The mean turnover intention score was 2.75 ± 1.06. This suggests there is a moderate intention to leave their place of employment among participants.

In comparison to norm scores, participants scored on the lower end of the average scale for vigour (3.33 ± 0.05), whereas for dedication (4.27 ± 0.04) and absorption (4.06 ± 0.04) participants’ scores reflected levels on the higher end of the average scale.

### Flourishing and job satisfaction associated with key workplace characteristics

Associations were identified between key workplace characteristics and intrapersonal flourishing and job satisfaction, but not work engagement and turnover intention (Table 2). Based on the confidence intervals no noteworthy associations were found between the other variables. As such data are presented in Table 2, but not elaborated on here.

*Role within the CTU* was associated with intrapersonal flourishing (95% CI 0.01, 0.08)*.* There was evidence of a positive association between working in a director, deputy, or senior management role (16.05 ± 0.40) and higher intrapersonal flourishing compared to those working in IT/Data (13.77 ± 0.32) (95% CI 0.71, 3.85) and Trial management (14.60 ± 0.18) (95% CI 0.04, 2.84).

*Salary* was positively associated with intrapersonal flourishing (95% CI 0.01–0.07). Most notably, those earning over £51,000 (15.85 ± 0.27) reported higher intrapersonal flourishing than those earning up to £30,999 (14.04 ± 0.37) (95% CI 0.57, 3.05) and those earning £41,000-£50,999 (14.92 ± 0.24) (95% CI 0.14, 2.35).

Having a *flexible working policy agreement in place* was positively associated with intrapersonal flourishing (95% CI 0.001, 0.04), with higher levels of intrapersonal flourishing in employees with a flexible working policy agreed (15.10 ± 0.18), compared to those without (14.40 ± 0.19). A similar association was identified with job satisfaction (95% CI 0.001, 0.04), with higher job satisfaction observed in employees with a flexible policy agreed (36.41 ± 0.36) compared to those without such an agreement (34.96 ± 0.42).

### The role of flourishing in job satisfaction

The variance inflation factors (VIFs) and tolerance factors for the predictor variables were no larger than 1.45 and no smaller than 1.00 respectively. Therefore, they did not contravene the threshold values for VIF of at least 5 and tolerance statistics of less than 0.2 that are used to suggest collinearity between independent variables.

Flourishing was associated with job satisfaction explaining 58.0% of the variance (R^2^ = 0.580, ΔR = 0.552, F = 132.26 *p* < 0.001), and turnover intention, explaining 44.5% of the variance (R^2^ = 0.445, ΔR = 0.434, F = 76.62, *p* < 0.001). Intrapersonal flourishing was the strongest predictor of both higher job satisfaction and lower turnover intention. No noteworthy associations were identified between flourishing and vigour, which accounted for 1.5% of the variance (R^2^ = 0.015, ΔR = 0.005, F = 1.50, *p* = 0.19), dedication, accounting for 0.9% of the variance (R^2^ = 0.009, ΔR = 0.006, F = 0.85, *p* = 0.51) and absorption, accounting for 1.8% of the variance (R^2^ = 0.018, ΔR = 3.81, F = 1.78, *p* = 0.114). As such, the individual predictive value of these measures of work engagement were not further explored, but are reported in Table 3.

**Table 3:** Regression analysis with job satisfaction measures used as dependent variables, defining workplace characteristics as predictor variables in step 1 and flourishing measures as predictor variables in step 2

	**Job Satisfaction**					**Turnover Intention**				
**Predictors**	**B**	**Β**	***t***	**Sig**	**CI**	**B**	**β**	***t***	**Sig**	**CI**
*Step 1*										
Salary	.71	.12	2.55	.01	-.41—.58	-.50	.291	−1.72	.09	−1.07—.07
Role	.09	.02	.34	.73	-.41—.58	-.25	.26	-.95	.34	-.76—.26
Flexi agreement	−1.36	-.11	−2.45	.02	−2.45—- .27	.35	.58	.60	.55	-.79—1.49
*Step 2*										
Interpersonal	.43	.23	6.50	< .001	.30—.56	-.08	.08	−1.05	.29	-.24-.07
Intrapersonal	1.29	.61	17.02	< .001	1.14 – 1. 44	−1.424	.090	−15.76	< .001	−1.60—−1.25
	**Vigour**					**Dedication**				
*Step 1*										
Salary	-.09	-.08	−1.63	.10	-.20—.02	-.02	-.02	-.39	.70	-.12—.08
Role	.01	.01	.12	.91	-.09—.10	-.00	-.00	-.03	.98	-.09—.09
Flexi agreement	.15	.06	1.36	.17	-.07—.36	.11	.05	1.14	.26	-.08—.31
*Step 2*										
Interpersonal	-.03	-.07	−1.32	.19	-.06—.01	-.03	-.08	−1.48	.14	-.06—.01
Intrapersonal	.03	.08	1.51	.13	-.01—.08	.00	.01	.15	.88	-.04—.04
	**Absorption**									
*Step 1*										
Salary	-.05	-.05	−1.12	.27	-.13—.04					
Role	.01	.01	.18	.86	-.07—.08					
Flexi agreement	.01	.01	.13	.90	-.16 – 1.8					
*Step 2*										
Interpersonal	-.04	-.15	−2.76	.01	-.07—-.01					
Intrapersonal	.02	.07	1.31	.19	-.01—.06					

### Pathways that lead to high flourishing at work

Using an inductive and deductive thematic analysis approach, three key themes (social elements, flexibility and autonomy, and meaning and achievement) were generated in relation to the ‘satisfaction’ question (Table 4), and two key themes (poor social elements, and poor environment) were generated in relation to the ‘dissatisfaction’ question (Table 5). A third theme (lack of growth) was identified only in the low flourishing scorers for the dissatisfaction question.

**Table 4:** Themes, Sub-themes and Example Quotes of Factors Contributing to Job Satisfaction in High Flourishing (HF) and Low Flourishing (LF) CTU Staff

Themes & Sub-themes	Participant Quotes
**Social Aspects**	
Feeling appreciated	*“People recognising work you've done and thank you/giving you a shout out—especially senior people. Also small things that make you feel like someone cares if you have a good day” (HF)* *“it helps if this [hard work] is recognised with a thank you or some tacit form of acknowledgment of the fact. An email to say thank you/ show gratitude of a task being achieved well is really rewarding” (LF)*
Positive interactions with colleagues	*“Contact with many intelligent and motivated individuals. My colleagues are lovely to work with” (LF)* *“I am lucky to work with some great colleagues and this is certainly what makes work for me more enjoyable. we are lucky that there is a fair amount of stability and a lot of our staff have been with the CTU for a number of years. The working environment is also pretty good at communicating and team working.” (HF)*
Positive interactions with senior managers	*"My line manager is very supportive but allows me to manage my own workload in the way that suits me best. He always praises good work and is appreciative of suggestions about how we could improve processes and procedures." (HF)* *“I feel my line manager is very supporting and understanding too and has a genuine desire for the team to grow and develop.” (LF)*
**Meaning and Achievement**	
Impactful work	*" The larger picture of the contribution our CTU makes towards clinical research provides a lot of satisfaction. It would be nice to receive feedback from further down the line about how our research studies have contributed to any change in clinical guidelines *etc*." (HF)* *“Feeling connected with patients, carers, the NHS. Seeing that research makes a difference to people's lives.” (LF)*
Achievement	*“Solving problems, achieving milestones, achieving objectives” (HF)* *“Achieving goals satisfies me at work—often I find it's hard to find tangible goals in all stages of ongoing research.” (LF)*
**Flexibility and Autonomy**	
Flexibility in location	*“It is very flexible with working location. I can be in the office or at home whenever I want and can change it whenever I need.” (HF)* *“[…] flexible home and part-time working to support difficult [carer] commitments” (LF)*
Autonomy	*“I am given a lot of freedom and independence to organise my work as best suits me, including hours, and I am not micromanaged. I feel like the CTU respects that we have lives outside work and allow us to balance that how we see fit.” (HF)* *“I am given a lot of freedom and independence to organise my work as best suits me, including hours, and I am not micromanaged.” (LF)*

**Table 5:** Themes, Sub-themes and Example Quotes of Factors Contributing to Job Dissatisfaction in High Flourishing (HF) and Low Flourishing (LF) CTU Staff

Themes & Sub-themes	Participant Quotes
**Poor environment**	
Heavy workload	*“The work load and pressures of the job can be quite overwhelming. Spinning so many plates at once working on several different trials at different stages. The strong team environment helps with this but burn out is a real concern.” (HF)* *“Workloads have shot up in recent years, and more and more expectations have piled on. Constant pressure to perform but little acknowledgement Constant additions to workloads with no practical consideration of what is possible in the available time: an expectation that you will just do it (i.e. you explain your concerns and nothing is done about them)” (LF)*
Limited flexibility	*“More flexibility on home/office work arrangement would be excellent. If the industry can accommodate full time home working, which may suit some people better (as I live far away and commute by train), why can't the CTU at universities do the same?” (LF)* *“Compulsory two days per week in the office is arbitrary and does not benefit either me, my colleagues or the work itself—it just means I have an onerous commute and lose 2/3 h extra of my day.” (HF)*
Lack of job security	*" Contract insecurity makes me very anxious I love a lot of what I do, but not knowing when/ if I'll be out of a job due to contracts is so offputting” (LF)* *“Since our funding situation has changed substantially, I now feel very insecure at work and sometimes wonder whether I would be better prioritising job security at this early stage (applying for work elsewhere) or if it's better to stick here and hope for the best.” (HF)*
**Poor social elements**	
Poor communication with senior managers	*“Not always transparent of why some decisions have been made causing unrest. Should be easy to rectify with communication but doesn't appear to happen.” (HF)* *“CTU doesn't listen to issues raised from the ground, and is generally dismissive of things that senior managers don't think up themselves. Some staff roles that are required for the CTU to function effectively are not recognised, and the quality of the work suffers as a consequence—and the people who have to pick up the pieces from chaotic management feel overworked.” (LF)*
Poor communication with colleagues	*“I also find communication lines with other research groups can be very lacking (but largely tribute this to them not understanding the role of a CTU, or data managemet/statistics tasks in general. I am frequently not engaged until well into the study)” (HF)* *“Anyone on the team who doesn't show willingness to communicate / teamwork to get things done.” (LF)*
Lack of appreciation/recognition	*“Feeling dismissed due to being in a 'professional services' role—I (and various members of my team) input into trial protocols, lead data management strategy, teach and sometimes publish our own methodology papers but often feel a lot less recognised in a trial team than [other] groups.” (HF)* *“No acknowledgment for work completed, we can work on studies for years and no feedback is provided so end up with the 'whats the point' attitude.” (LF)*
**Growth**	
Personal/Professional Development	*“I don't feel my skills and knowledge are utilised enough and personal development feels stunted. This means I do not feel any sense of joy with work at the moment even when achieving really good results.” (LF)*
Career Progression	*“Promotion criteria are faculty (maybe even university) wide and don't always feel applicable to CTU staff with limited advice on how these can be met.” (LF)*

The themes were the same across individuals in the high interpersonal (*n* = 104) and intrapersonal group (*n* = 82), as such the themes are presented together to represent a group of high flourishers (total *n* = 186). Similarly, the themes were the same across individuals in the low interpersonal (*n* = 94) and intrapersonal (*n* = 89) group and both groups are presented together as low flourishers (*n* = 183). Any noteworthy differences *within* the themes, between those scoring high in interpersonal/intrapersonal flourishing and low interpersonal/intrapersonal flourishing scorers are presented where applicable.

### High job satisfaction

The factors contributing to increased job satisfaction, as identified by both high and low flourishing participants, were social elements, flexibility and autonomy, and meaning and achievement.

#### Social Elements

Social elements encompassed positive interactions with colleagues, supportive senior managers including, line managers and other senior staff, as well as feeling appreciated and recognised for their expertise and contributions by all. In terms of *colleagues*, participants reported satisfaction with colleagues who value each other, offer help and problem solving when needed, as well as having opportunities for socialising outside work. This contributed to a sense of belonging within the team/CTU. In terms of *senior managers*, participants appreciated feeling well-supported by seniors who actively listen and attempt to address any issues raised, and this increased their job satisfaction and motivation in the workplace.

#### Flexibility and Autonomy

Flexible working patterns, as well as hybrid working, was associated with high job satisfaction. Participants valued not having to adhere to a fixed-office day mandate and appreciated a personalised approach. Additionally, participants valued autonomy in managing their workload. Flexibility and autonomy were more frequently identified as leading to high job satisfaction by those scoring high in interpersonal flourishing compared to those scoring high in intrapersonal flourishing and those scoring low in interpersonal/intrapersonal flourishing.

#### Meaning and Achievement

High flourishing participants wrote about the importance of their work making a difference; contributions to the wider public was described as one of the key factors that made high flourishing employees feel satisfied at work. A *sense of achievement* derived from reaching study aims to time and target was also associated with high job satisfaction, although it was noted that this was not always possible due to heavy workloads. Meaning and achievement was more evident in the narratives of those scoring high in intrapersonal flourishing and low in interpersonal/intrapersonal flourishing compared to those scoring high on interpersonal flourishing.

### Low job satisfaction

Several *environmental* factors, such as heavy workload and lack of job security, and *poor social elements* contributed to low job satisfaction in both high and low flourishing scorers.

#### Poor Environment

Participants shared that their *workload* was often overwhelming due to restricted resources, including time and budget and high staff turnover. This increased work pressures and contributed to a lack of feeling of achievement. Inefficient processes, excessive documentation, lack of job security and low pay relative to demands and experience, also contributed to participants’ dissatisfaction with the workplace. Poor environmental factors were frequently identified as leading to low job satisfaction particularly by participants scoring high in interpersonal/intrapersonal flourishing.

#### Poor Social Elements

Lack of appreciation for hard work and expertise was frequently mentioned under reasons for job dissatisfaction. Some participants noted that there was lack of recognition for their role, which was often not recognised as academic. Poor communication with senior managers and a lack of effective leadership, led participants to feel poorly supported and undervalued. Participants also shared their frustration with the inconsistent treatment of staff within their CTU. Other social elements linked to workplace dissatisfaction were in relation to poor interactions with colleagues and external members of trial teams. Poor social elements in the workplace, specifically poor communication with senior managers and low team morale, were frequently identified as leading to low job satisfaction, particularly by participants scoring low in interpersonal/intrapersonal flourishing.

#### Lack of Growth

Having limited opportunities and an unclear pathway to career progression, limited skill development and/or time to pursue them, were identified as factors contributing to job dissatisfaction; particularly by those scoring low in interpersonal/intrapersonal flourishing.

## Discussion

With the aim of gaining insight into the relationship between flourishing and job satisfaction (assessed by turnover intention, work engagement, and job satisfaction measures) among CTU staff a national survey of employees working in UKCRC-registered CTUs was conducted.

### Job satisfaction in CTU staff

Participants exhibited moderate intentions to leave their workplace, reflecting a national concern regarding the recruitment and retention of experienced clinical research staff [4, 17]. In addition, a high proportion of the sample indicated that they were often thinking about getting another job that would better suit their personal needs. If no measures are taken to reverse this trend, there will be significant implications for the operational efficiency of CTUs. It could further pose challenges to meeting government mandates aimed at enhancing the UK's healthcare research capabilities global health crises [2]. CTU staff reported average levels of job satisfaction, with lower levels reported by those without a flexible working policy agreement and earning a lower salary. Many participants cited heavy workload and associated fatigue as one of the key factors leading to job dissatisfaction. Average job satisfaction and low-average energy levels (vigour) align with CTU staff being only partially committed to their CTU. Together, these findings suggest that intentions to leave may stem from multifaceted and intricate factors which may reflect the multi-dimensional working environment of a CTU. By examining the interplay between flourishing, workplace characteristics and job satisfaction metrics, and by integrating insights from both psychometric assessments and qualitative responses, we can gain a deeper understanding of how to foster a positive organisational culture. These insights may enhance employee engagement and bolster commitment within the CTU.

### Flourishing in CTU staff

In comparison to other research using the EWWS, participants reported slightly lower levels of workplace flourishing than other samples. In this study we report comparisons of our UK sample with employees working in the Netherlands [47], Saudi Arabia [48], and against a large sample with geographical location not reported [27]. It should be noted that it was not possible to identify the specific nature of these employees work or job role. Indeed, it was challenging to make direct comparisons with other employees as there is a lack of peer-reviewed papers which transparently report appropriate descriptive statistics (e.g., means and standard deviations) to describe their samples levels of flourishing. It is hoped that by reporting sample sizes and descriptive statistics by job role, we can support future comparative work in this area as interest in the field of positive psychology grows. This may be important since we identified differences in levels of intrapersonal flourishing (encompassing value, purpose, control, and autonomy [27]), depending upon job role, salary, and having a flexible working arrangement. Free-text responses revealed why these workplace characteristics may affect intrapersonal flourishing, and these are subsequently discussed.

Monotonous work and that which participants struggled to see the wider impact of, was linked with dissatisfaction and low flourishing. Believing one’s role is unrecognised and feeling underpaid was perceived as a lack of appreciation and appeared to reduce participants motivation to develop professionally. Lower flourishing in lower salaried roles could also be explained by the limited autonomy afforded to more junior employees over the tasks assigned to them and their workloads. In contrast, those working in senior management roles such as senior trial/data/programming managers, operational directors, head of CTUs and deputies, experienced higher intrapersonal flourishing. It is possible that those working in more senior roles perceive greater control afforded by their position, higher monetary rewards, and benefit from the greater impact of their contributions to research. These elements, meaningful work, perceived control and autonomy, are elements of intrapersonal flourishing [27, 49].

It should be noted that it is not possible to discern whether high intrapersonal flourishing is the driving force leading to career development in CTU staff, or if achieving a certain seniority level is what boosts intrapersonal flourishing, influenced by the factors outlined above [50]. Employees who experience high levels of flourishing may be more likely to seek out and be selected for senior positions due to their positive attributes, such as motivation, resilience, and leadership skills [51]. We believe the relationship between intrapersonal flourishing and senior positions is likely bidirectional, with each influencing and reinforcing the other to some extent. This could be further explored in a longitudinal evaluation of CTU staff.

Another important characteristic of the workplace linked with flourishing was flexible working. Respondents linked having a flexible working policy agreement in place with their line manager with trust, respect, and autonomy; a key aspect of intrapersonal flourishing [27]. A flexible working policy arrangement could support a sense of autonomy and perceived control over the working environment [49, 52]. Hybrid and flexible working reduced stress and increased work-life balance, contributing to greater job satisfaction. This is consistent with recent research highlighting that the freedom to independently balance work and other life demands, through flexible working, may lead to better work-life satisfaction [4], improved work engagement and increased commitment to the workplace [53, 54].

### Relationship between flourishing and job satisfaction measures

Both interpersonal and intrapersonal flourishing predicted unique variance in job satisfaction and turnover intention when controlling for workplace characteristics. Specifically, intrapersonal flourishing (e.g., value, control, autonmy), emerged as the strongest predictor of both job satisfaction and turnover intention. Previous research, although limited, supports this finding and proposes that intrapersonal flourishing is a better predictor of both workplace and overall wellbeing compared to other measures [55]. Nonetheless, interpersonal flourishing (e.g., social connections) should not be overlooked. Supportive relationships helped employees feel listened to, increasing their sense of being valued, and contributing to them feeling able to initiate change, thus increasing their perceived control over their environment. Positive interactions with their colleagues, both at work and through other social activities outside working hours, increased employees’ sense of belonging. Participants shared that not only feeling well supported by colleagues, but also helping colleagues, made them feel satisfied at work, showing that a culture of reciprocity can have benefits for all involved.

The unique role of flourishing in job satisfaction and turnover intention in CTU staff is important. Whilst it is not always possible or practical to influence factors such as salary, or working in a senior role, it may be possible to mitigate against poor job satisfaction and commitment to the workplace by improving flourishing. This can be achieved primarily by increasing perceptions of the value and meaning that an individual brings to a CTU.

### Model of workplace flourishing in CTU staff

**Fig. 1:**
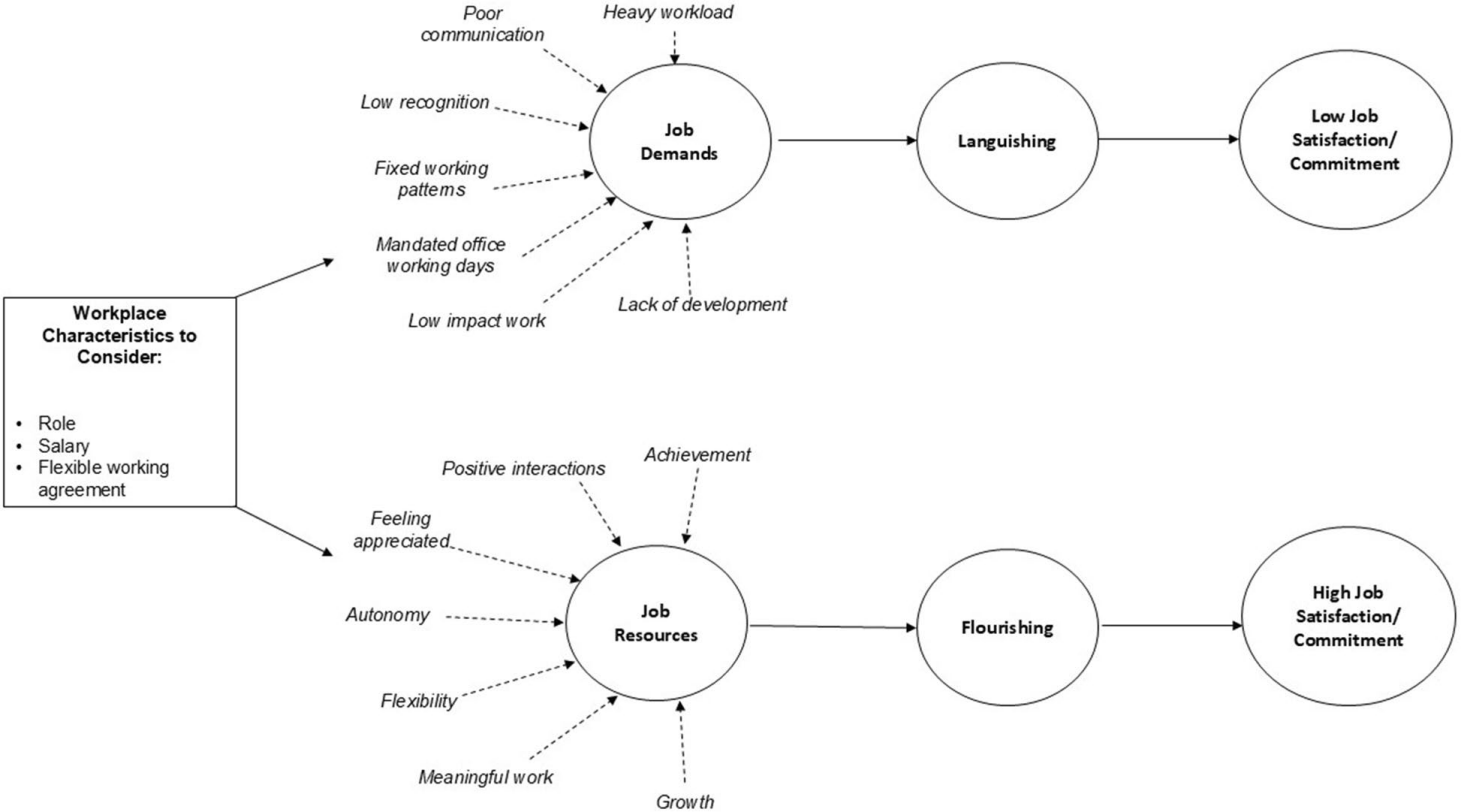
How a CTU working environment relates to flourishing and job satisfaction

Figure 1 provides a graphical illustration of our results. This figure illustrates how workplace wellbeing could be conceptualised within CTUs, by combining a flourishing (or eudaimonic) model of wellbeing with the JD-R model. This approach enables us to combine a more novel perspective of positive wellbeing (flourishing) within the well-established and widely implemented JD-R model. In doing so, we not only hope to facilitate acceptance and uptake of our approach, but also, we can begin to identify pathways/strategies to promote positive wellbeing, as well as identify aspects of the working environment which may place employees at risk of experiencing low-flourishing or languishing.. As outlined in Fig. 1, a flourishing workplace was associated with a positive social environment, consisting of positive interactions with colleagues and trusting relationships with senior managers who strive to create a sense of belonging with fair decision making. Employees’ efforts are recognised, they have flexibility and control over their working environment and workloads, as well as the opportunity to contribute to meaningful work. In addition, secure contracts, and the opportunity to grow and achieve senior roles/higher salaries are markers of a flourishing workplace.

### Implications

The implementation of a flourishing model within UK CTUs could enhance employee resilience. This can be achieved by creating a supportive environment that fosters a sense of belonging, allows for flexibility and autonomy, and ensures that staff can see their work is meaningful. Strengthening social relationships and team collaboration can further boost teamwork and problem-solving, which is crucial given the unpredictable challenges inherent in trial work. A work environment where CTU staff can flourish, may better equip them—mentally and emotionally—to handle the high-pressure demands of their jobs. This, in turn, can lead to greater commitment and sustained effort over time, lower turnover, and the retention of high levels of expertise and experience within the CTU.

### Strengths and limitations

The study findings are strengthened by the large sample size obtained with participants from 52 UKCRC-registered CTUs. Although most participants were female, this reflects the CTU workforce gender balance [56]. Furthermore, most (88%) of participants were White, with 6% belonging to Asian/Asian British (6%), but this broadly aligns with the UK population characteristics [57].

A strength of the study lies in its utilisation of standardised psychometric assessments to gauge levels of flourishing, job satisfaction, and turnover intention. Whilst the survey approach enabled us to formally evaluate levels of workplace wellbeing and job satisfaction in CTU staff working in the UK, at scale, it is possible that the participants provided biased answers. To reduce this, survey responses were collected anonymously, and the confidentiality and anonymity procedures were emphasised. The survey method also supported rapid, real-time data collection enabling timely insights. This came at an important period of change in the working environment as many organisations navigated the transition to hybrid working following the COVID-19 health pandemic. Indeed, it is important to recognise that the data collection period is likely to have influenced the findings of this study, future comparisons over different time periods and societal shifts are recommended. Nonetheless, although the survey approach is advantageous, as previously mentioned, cause-effect conclusions cannot be established based on the data obtained.

This study marks a pivotal initial stride in constructing a simple structured framework for conceptualising wellbeing in CTU staff, guiding hypothesis development, shaping research design, and facilitating the practical application of findings. Future work is required to refine and evaluate this model and develop associated guidance to monitor and improve workplace wellbeing within CTUs. Investing in this may prove useful not only for development of the healthcare research workforce, but also for understanding how best to protect the workplace wellbeing of more frontline healthcare workers where recruitment and retention issues are challenging efficient delivery of services.

## Conclusions

CTU staff reported average levels of job satisfaction and work engagement, lower levels of flourishing in comparison to other published research using the same scale, and a moderate intention to leave their place of employment. Higher levels of flourishing were predictive of job satisfaction and turnover intention, highlighting the importance of promoting flourishing within CTUs. Flourishing employees are not just satisfied with their jobs but are thriving in their roles. They are more likely to be committed to their CTU because they perceive their job as meaningful, and they feel valued and supported by colleagues and senior managers. By providing employees with the job resources needed to mitigate the associated work demands of clinical trials, a working environment can be developed which would allow CTU staff to flourish. This is likely to have implications for employees’ job satisfaction, work engagement and commitment to their organisation and could improve organisational efficiency. Prioritising efforts to understand and promote wellbeing and job satisfaction of CTU staff has potential implications for equipping the UK to lead in the development of healthcare innovations to address global healthcare challenges.

## Supplementary Information

Supplementary Material 1.

Supplementary Material 2.

Supplementary Material 3.

## Data Availability

The data are available from the authors upon reasonable request and with the permission of the study team.

## Abbreviations

CTU: Clinical Trial Unit
UKCRC: UK Clinical Research Collaboration
UKTMN: UK Trial Managers' Network
TMRP: Trials and Methodology Research Partnership
EWWS: Eudaimonic Workplace Wellbeing Scale
UWES: Utrecht Work Engagement Scale
JSS: Job Satisfaction Scale
TIS: Turnover Intention Scale
J-D R: Job-Demands and Resources
VIF: Variance Inflation Factors
CI: Confidence Intervals

## References

[1] National Health Service. Science in healthcare: Delivering the NHS Long Term Plan. The Chief Scientific Officer’s strategy. 2020. https://www.england.nhs.uk/wp-content/uploads/2020/03/science-in-healthcare-delivering-the-nhs-long-term-plan.pdf. Accessed 25 April 2024.

[2] Department of Health and Social Care. The Future of Clinical Research Delivery: 2022 to 2025 implementation plan. 2022. https://www.gov.uk/government/publications/the-future-of-uk-clinical-research-delivery-2022-to-2025-implementation-plan/the-future-of-clinical-research-delivery-2022-to-2025-implementation-plan. Accessed 30 April 2024.

[3] Hall CL, Sanderson C, Brown B, et al. Opportunities and challenges of delivering digital clinical trials: lessons learned from a randomised controlled trial of an online behavioural intervention for children and young people. Trials. 2020;21(1):1–13. 33298127 10.1186/s13063-020-04902-1 PMC7724811

[4] Mitchell E, Goodman K, Wakefield N, et al. Clinical trial management: a profession in crisis? Trials. 2022;23(1):1–4. 35477835 10.1186/s13063-022-06315-8 PMC9044377

[5] Fordyce CB, Rose MT, Pierre C, et al. Trends in clinical trial investigator workforce and turnover: An analysis of the US FDA 1572 BMIS database. Contemp Clinic Trials Communic. 2019;15:100380. 10.1016/j.conctc.2019.100380 PMC6536616 31193608

[6] Fordyce CB, Malone K, Forrest A, et al. Improving and sustaining the site investigator community: recommendations from the clinical trials transformation initiative. Contemp Clinic Trials Communic. 2019;16:100462. 10.1016/j.conctc.2019.100462 PMC6831713 31701037

[7] Lai J, Forney L, Brinton DL, Simpson KN. Drivers of start-up delays in global randomized clinical trials. Therap Innov Reg Sci. 2021;55(1):212–27. 10.1007/s43441-020-00207-2 PMC7505220 32959207

[8] Duley L, Gillman A, Duggan M, et al. What are the main inefficiencies in trial conduct: a survey of UKCRC registered clinical trials units in the UK. Trials. 2018;19(1):1–7. 29310685 10.1186/s13063-017-2378-5 PMC5759880

[9] Department for Business, Energy and Industrial Stategy. R&D People and Culture Strategy: People at the heart of R&D. 2021. https://assets.publishing.service.gov.uk/government/uploads/system/uploads/attachment_data/file/1004685/r_d-people-culture-strategy.pdf. Accessed 5^th^ March 2024.

[10] Gayvert KM, Madhukar NS, Elemento O. A data-driven approach to predicting successes and failures of clinical trials. Cell Chemic Biol. 2016;23(10):1294–301. 10.1016/j.chembiol.2016.07.023 PMC5074862 27642066

[11] Elkin ME, Zhu X. Understanding and predicting COVID-19 clinical trial completion vs. cessation. Plos one. 2021;16(7):e0253789. 34252108 10.1371/journal.pone.0253789 PMC8274906

[12] Tognoni G, Alli C, Avanzini, et al. Randomised clinical trials in general practice lessons from a failure. BMJ. 1991;303(6808):969. 1954424 10.1136/bmj.303.6808.969 PMC1671349

[13] Fogel DB. Factors associated with clinical trials that fail and opportunities for improving the likelihood of success: a review. Contemp Clinic Trials Communic. 2018;11:156–64. 10.1016/j.conctc.2018.08.001 PMC6092479 30112460

[14] Thoma A, Farrokhyar F, McKnight A, Bhandari M. How to optimize patient recruitment. Canadian J Surge. 2010;53(3):205. PMC2878987 20507795

[15] Briel M, Elger BS, McLennan, et al. Exploring reasons for recruitment failure in clinical trials: a qualitative study with clinical trial stakeholders in Switzerland, Germany, and Canada. Trials. 2021;22(1):1–13. 34823582 10.1186/s13063-021-05818-0 PMC8613940

[16] BDO. 2022/2023 Clinical Research Organization Insights Report: Managing Talent and Pay in a Competitive Market and Volatile Economy. 2023. https://www.bdo.com/getmedia/a6fb62d9-36f2-46d9-a053-deeffc84ca30/TAX_GES_2022-2023-BDO-CRO-Insights-Report.pdf?ext=.pdf. Accessed 10^th^ April 2024.

[17] Sun G, Dizon DS, Szczwpanek, et al. Crisis of the Clinical Trials Staff Attrition After the COVID-19 Pandemic. JCO Oncol Pract. 2023;19(8):533–6. 10.1200/OP.23.00152 PMC10424897 37285550

[18] Dizon DS, Szczwpanek CM, Petrylak DP, et al. National impact of the COVID-19 pandemic on clinical trial staff attrition: Results of the SWOG Cancer Research Network Survey of Oncology Research Professionals. J Clinic Oncol. 2022;40:16. 10.1200/JCO.2022.40.16_suppl.11049.

[19] Freel SA, Snyder DC, Bastarache K, et al. Now is the time to fix the clinical research workforce crisis. Clinical Trials. 2023;20(5):457–62. 37264897 10.1177/17407745231177885 PMC10504806

[20] Lorenc A, Rooshenas L, Conefrey C. Non-COVID-19 UK clinical trials and the COVID-19 pandemic: impact, challenges and possible solutions. Trials. 2023;24(1):424. 37349850 10.1186/s13063-023-07414-w PMC10286467

[21] Bakker AB, Demerouti E, De Boer E, Schaufeli WB. Job demands and job resources as predictors of absence duration and frequency. J Vocat Behav. 2003;62(2):341–56.

[22] Hogg B, Medina JC, Gardoki-Souto, et al. Workplace interventions to reduce depression and anxiety in small and medium-sized enterprises: A systematic review. J Affective Disord. 2021;290:378–86. 10.1016/j.jad.2021.04.071 34082284

[23] Yunus WMAWM, Musiat P, Brown JS. Systematic review of universal and targeted workplace interventions for depression. Occu Environ Med. 2018;75(1):66–75. 10.1136/oemed-2017-104532 29074553

[24] Keyes CL, Shmotkin D, Ryff CD. Optimizing well-being: the empirical encounter of two traditions. J Person Soc Psychol. 2002;82(6):1007. 12051575

[25] Ryff CD, Boylan JM, Kirsch JA. Eudaimonic and hedonic wellbeing: An integrative perspective with linkages to sociodemographic factors and health. In: Lee MT, Kubzansky LD, VanderWeele TJ, editors. Measuring well-being: Interdisciplinary perspectives from the social sciences and humanities. Oxford: Oxford University Press; 2021. 10.1093/oso/9780197512531.003.0005.

[26] Ryff CD, Keyes CLM. The structure of psychological well-being revisited. J Personal Social Psychol. 1995;69(4):719. 10.1037//0022-3514.69.4.719 7473027

[27] Bartels AL, Peterson SJ, Reina CS. Understanding well-being at work: Development and validation of the eudaimonic workplace well-being scale. PLoS ONE. 2019;14(4):e0215957. 31022285 10.1371/journal.pone.0215957 PMC6483236

[28] Lambert EG, Minor KI, Wells JB, Hogan NL. Social support’s relationship to correctional staff job stress, job involvement, job satisfaction, and organizational commitment. Soc Sci J. 2016;53(1):22–32.

[29] Penconek T, Tate K, Bernardes A, et al. Determinants of nurse manager job satisfaction: A systematic review. International J Nurs Stud. 2021;118:103906. 10.1016/j.ijnurstu.2021.103906 33765624

[30] Jolly PM, Kong DT, Kim KY. Social support at work: An integrative review. J Organiz Behav. 2021;42(2):229–51.

[31] Fazio J, Gong B, Sims R, Yurova Y. The role of affective commitment in the relationship between social support and turnover intention. Manag Dec. 2017;55(3):512–25.

[32] Fong LHN, Chui PMW, Cheong ISC, Fong DKC. Moderating effects of social support on job stress and turnover intentions. J Hospit Market Manag. 2018;27(7):795–810.

[33] Velando-Soriano A, Ortega-Campos E, Gomez-Uriquiza L, et al. Impact of social support in preventing burnout syndrome in nurses: A systematic review. Japan J Nurs Sci. 2020;17(1):e12269. 31617309 10.1111/jjns.12269

[34] Tsen MK, Gu M, Tan CM, Goh SK. Effect of flexible work arrangements on turnover intention: does job independence matter? Int J Sociol. 2021;51(6):451–72.

[35] Gašić D, Berber N. The Mediating Role of Employee Engagement in the Relationship between Flexible Work Arrangements and Turnover Intentions among Highly Educated Employees in the Republic of Serbia. Behav Sci. 2023;13(2):131. 36829360 10.3390/bs13020131 PMC9952613

[36] Tsen MK, Gu M, Tan CM, Goh SK. Does flexible work arrangements decrease or increase turnover intention? A comparison between the social exchange theory and border theory. Int J Sociol Soc Pol. 2022;42(11–12):962–83.

[37] Schaufeli WB, Bakker AB. Job demands, job resources, and their relationship with burnout and engagement: A multi-sample study. J Organiz Behav. 2004;25(3):293–315.

[38] Bakker AB, Demerouti E. The job demands-resources model: State of the art. J Managerial Psychol. 2007;22(3):309–28.

[39] Demerouti E, Bakker AB, Nachreiner F, Schaufeli WB. The job demands-resources model of burnout. J Appl Psychol. 2001;86(3):499. 11419809

[40] Mora V, Colantuono S, Fanali C, et al. Clinical research coordinators: Key components of an efficient clinical trial unit. Contemp Clinic Trials Commun. 2023;21(32):101057. 10.1016/j.conctc.2023.101057 PMC9898615 36747989

[41] Schaufeli WB, Salanova M, Gonzales-Roma V, et al. The measurement of engagement and burnout: A two sample confirmatory factor analytic approach. J Happi Stud. 2002;3(1):71–92.

[42] Van Saane N, Sluiter JK, Verbeek JH, Frings-Dresen MH. Reliability and validity of instruments measuring job satisfaction—a systematic review. Occup Medic. 2003;53(3):191–200. 10.1093/occmed/kqg038 12724553

[43] Bothma CF, Roodt G. The validation of the turnover intention scale. SA J Hum Resour Manag. 2013;11(1):1–12.

[44] Du Plooy J, Roodt G. Work engagement, burnout and related constructs as predictors of turnover intentions. SA J Indust Psychol. 2010;36(1):1–13.

[45] Proudfoot K. Inductive/deductive hybrid thematic analysis in mixed methods research. J Mixed Methods Res. 2023;17(3):308–26.

[46] Macdonald S, Maclntyre P. The generic job satisfaction scale: Scale development and its correlates. Employee Assistance Quarterly. 1997;13(2):1–16.

[47] Vries AD, Broks VM, Bloemers W, Kuntze J, De Vries RE. Self-, other-, and meta-perceptions of personality: Relations with burnout symptoms and eudaimonic workplace well-being. PLoS ONE. 2022;17(7):e0272095. 35901041 10.1371/journal.pone.0272095 PMC9333331

[48] Al Kahtani NS, Mm S. A study on how psychological capital, social capital, workplace wellbeing, and employee engagement relate to task performance. Sage Open. 2022;12(2):2158244022109501.

[49] Soren A, Ryff CD. Meaningful work, well-being, and health: enacting a eudaimonic vision. Int J Environ Res Public Health. 2023;20(16):6570. 37623156 10.3390/ijerph20166570 PMC10454804

[50] Boyce CJ, Oswald AJ. Do people become healthier after being promoted? Health economics. 2012;21(5):580–96. 21506192 10.1002/hec.1734

[51] Zhang Y, Wang F, Cui G, Qu J, Cheng Y. When and why proactive employees get promoted: A trait activation perspective. Curr Psychol. 2023;42(36):31701–12. 10.1007/s12144-022-04142-3 PMC9794477 36590012

[52] Mikus J, Rieger J, Grant-Smith D. Eudaemonic design to achieve well-being at work, wherever that may be, in Ergonomics and Business Policies for the Promotion of Well-Being in the Workplace. IGI Global. 2022;1–32.

[53] Choi S. Flexible work arrangements and employee retention: A longitudinal analysis of the federal workforces. Public Personnel Management. 2020;49(3):470–95. 10.1177/0091026019886340.

[54] Richman AL, Civian JT, Shannon LL, et al. The relationship of perceived flexibility, supportive work–life policies, and use of formal flexible arrangements and occasional flexibility to employee engagement and expected retention. Community, Work Family. 2008;11(2):183–97.

[55] Rothausen TJ, Henderson KE. Meaning-based job-related well-being: exploring a meaningful work conceptualization of job satisfaction. J Bus Psychol. 2019;34:357–76.

[56] Roberts I. Women’s work in UK clinical trials is undervalued. The Lancet. 2018;392(10149):732. 10.1016/S0140-6736(18)31540-X 30191824

[57] Office for National Statistics. https://www.ons.gov.uk/peoplepopulationandcommunity/culturalidentity/ethnicity. Accessed 12^th^ March 2024.

