# Supplementary material for: Flourishing and job satisfaction in employees working in UK clinical trial units: a national cross-sectional survey
Source: BMC Health Serv Res. 2024 Dec 2;24:1522. doi: 10.1186/s12913-024-11986-x (PMC11610179; doi:10.1186/s12913-024-11986-x)
Supplement: Supplementary file 1 — Supplementary Material 1. [file 12913_2024_11986_MOESM1_ESM.docx]

FACTS - Flourishing As Clinical Trial Staff - Survey

Demographics

Please select the gender you identify with:

Please select the ethnic group you identify with:

If you selected Other, please specify:

Please select your age category:

Please select which UKCRC Clinical Trials Unit (CTU) you currently work at: *Your answer to this question will****not****be linked to your demographic data. The data will be analysed in groups (e.g. based on region).*

How long have you been working at your current CTU? *(Please round to the nearest year).*

What type of contract are you on?

If you selected Other, please specify:

Do you work full time or part time?

What salary scale are you on? *This data will be used e.g. to discern seniority within your workplace.*

What is your CTU's policy on home/office-based working?

If you selected Other, please specify:

What is your **preferred** home/office-based working policy?

If you selected Other, please specify:

Do you have flexible working arrangements agreed with your line manager? *(Flexible working arrangements may include working over different days, times or any specific alternate arrangements to what your CTU policies are).*

What best describes your main job role?

If you selected Other, please specify:

Utrecht Work Engagement Scale

**The following 9 statements are about how you feel at work. Please read each statement carefully and decide if you ever feel this way about your job.**

**If you have never had this feeling, select 'Never'. If you have had this feeling, indicate how often you felt it by selecting the statement that best describes how frequently you feel that way.**

Please remember, your data will be kept anonymous.

|  | Never | Almost never (a few times a year or less) | Rarely (once a month or less) | Sometimes (a few times a month or less) | Often (once a week) | Very often (a few times a week) | Always (every day) |
| --- | --- | --- | --- | --- | --- | --- | --- |
| At my work, I feel bursting with energy |  |  |  |  |  |  |  |
| At my job, I feel strong and vigorous |  |  |  |  |  |  |  |
| I am enthusiastic about my job |  |  |  |  |  |  |  |
| My job inspires me |  |  |  |  |  |  |  |
| When I get up in the morning, I feel like going to work |  |  |  |  |  |  |  |
| I feel happy when I am working intensely |  |  |  |  |  |  |  |
| I am proud of the work that I do |  |  |  |  |  |  |  |
| I am immersed in my work |  |  |  |  |  |  |  |
| I get carried away when I am working |  |  |  |  |  |  |  |

Eudaimonic Workplace Wellbeing Scale

**This portion of the survey consists of a number of statements that may describe how you feel within your workplace. Please indicate your agreement with the following statements:**

|  | Strongly disagree | Disagree | Neither agree nor disagree | Agree | Strongly agree |
| --- | --- | --- | --- | --- | --- |
| Among the people I work with, I feel there is a sense of brotherhood/sisterhood |  |  |  |  |  |
| I feel close to the people in my work environment |  |  |  |  |  |
| I feel connected to others within the work environment |  |  |  |  |  |
| I consider the people I work with to be my friends |  |  |  |  |  |
| I am emotionally energized at work |  |  |  |  |  |
| I feel that I have a purpose at my work |  |  |  |  |  |
| My work is very important to me |  |  |  |  |  |
| I feel I am able to continually develop as a person in my job |  |  |  |  |  |

Job Satisfaction Scale

**For each statement please select your degree of agreement.**

Please remember, your data will be kept anonymous.

|  | Strongly disagree | Disagree | Don’t know | Agree | Strongly agree |
| --- | --- | --- | --- | --- | --- |
| I receive recognition for a job well done |  |  |  |  |  |
| I feel close to the people at work |  |  |  |  |  |
| I feel good about working at this CTU |  |  |  |  |  |
| I feel secure about my job |  |  |  |  |  |
| I believe my senior managers are concerned about me |  |  |  |  |  |
| On the whole, I believe work is good for my physical health |  |  |  |  |  |
| My wages are good |  |  |  |  |  |
| All my talents and skills are used at work |  |  |  |  |  |
| I get along with my line manager |  |  |  |  |  |
| I feel good about my job |  |  |  |  |  |

Turnover Intention Scale

Please read each question and indicate your response using the response options provided. Please consider how you have felt over the past 6 months.

Please answer each question **'during the past 6 months'**.

*Please remember, your data will be kept anonymous.*

How often have you considered leaving your job?

|  | 1 | 2 | 3 | 4 | 5 |  |
| --- | --- | --- | --- | --- | --- | --- |
| Never |  |  |  |  |  | Always |

How satisfying is your job in fulfilling your personal needs?

|  | 1 | 2 | 3 | 4 | 5 |  |
| --- | --- | --- | --- | --- | --- | --- |
| Very satisfying |  |  |  |  |  | Totally dissatisfying |

How often are you frustrated when not given the opportunity at work to achieve your personal work-related goals?

|  | 1 | 2 | 3 | 4 | 5 |  |
| --- | --- | --- | --- | --- | --- | --- |
| Never |  |  |  |  |  | Always |

How often do you dream about getting another job that will better suit your personal needs?

|  | 1 | 2 | 3 | 4 | 5 |  |
| --- | --- | --- | --- | --- | --- | --- |
| Never |  |  |  |  |  | Always |

How likely are you to accept another job at the same compensation level should it be offered to you?

|  | 1 | 2 | 3 | 4 | 5 |  |
| --- | --- | --- | --- | --- | --- | --- |
| Highly unlikely |  |  |  |  |  | Highly likely |

How often do you look forward to another day at work?

|  | 1 | 2 | 3 | 4 | 5 |  |
| --- | --- | --- | --- | --- | --- | --- |
| Always |  |  |  |  |  | Never |

What is it about your working environment (e.g., things you do or things your CTU does) that makes you feel**satisfied** at work? *Required*

What is it about your working environment (e.g., things you do or things your CTU does) that makes you feel **dissatisfied** at work? *Required*
