# Supplementary material for: Flourishing and job satisfaction in employees working in UK clinical trial units: a national cross-sectional survey
Source: BMC Health Serv Res. 2024 Dec 2;24:1522. doi: 10.1186/s12913-024-11986-x (PMC11610179; doi:10.1186/s12913-024-11986-x)
Supplement: Supplementary file 2 — Supplementary Material 2. [file 12913_2024_11986_MOESM2_ESM.docx]

**Supplementary Material 2: Flourishing and Job Satisfaction in Employees Working in UK Clinical Trial Units: A National Cross-sectional Survey**

| **Correlation Matrix 1** | | | | | | | |
| --- | --- | --- | --- | --- | --- | --- | --- |
|  | | UWES1.Vig | UWES2.Vig | UWES3.Ded | UWES4.Ded | UWES5.Vig | UWES6.Abs |
| Correlation | UWES1.Vig | 1.000 | .817 | .649 | .620 | .626 | .539 |
|  | UWES2.Vig | .817 | 1.000 | .648 | .646 | .633 | .512 |
|  | UWES3.Ded | .649 | .648 | 1.000 | .819 | .664 | .582 |
|  | UWES4.Ded | .620 | .646 | .819 | 1.000 | .672 | .558 |
|  | UWES5.Vig | .626 | .633 | .664 | .672 | 1.000 | .600 |
|  | UWES6.Abs | .539 | .512 | .582 | .558 | .600 | 1.000 |
|  | UWES7.Ded | .493 | .538 | .650 | .651 | .576 | .490 |
|  | UWES8.Abs | .465 | .521 | .596 | .603 | .526 | .408 |
|  | UWES9.Abs | .350 | .343 | .350 | .405 | .347 | .304 |
|  | EWWS1.Inter | .000 | -.020 | -.031 | -.097 | -.076 | -.095 |
|  | EWWS2.Inter | -.048 | -.039 | -.045 | -.106 | -.059 | -.101 |
|  | EWWS3.Inter | -.024 | -.005 | -.001 | -.057 | -.073 | -.075 |
|  | EWWS4.Inter | .035 | -.002 | -.002 | -.067 | -.018 | -.060 |
|  | EWWS5.Intra | .039 | .026 | -.028 | -.046 | -.019 | -.067 |
|  | EWWS6.Intra | .066 | .047 | .019 | -.012 | -.053 | -.032 |
|  | EWWS7.Intra | .017 | .018 | .001 | .007 | -.026 | -.003 |
|  | EWWS8.Intra | .057 | .028 | -.004 | -.034 | -.004 | -.003 |
|  | JSS1 | .092 | .100 | .076 | .032 | .020 | -.007 |
|  | JSS2 | -.019 | -.011 | -.038 | -.060 | -.069 | -.120 |
|  | JSS3 | .023 | .013 | .018 | -.028 | -.046 | -.085 |
|  | JSS4 | -.042 | -.058 | -.015 | -.040 | -.075 | -.117 |
|  | JSS5 | .021 | .015 | -.022 | -.059 | -.025 | -.083 |
|  | JSS6 | .049 | .030 | .032 | -.035 | .022 | -.031 |
|  | JSS7 | .042 | .014 | -.006 | -.036 | -.019 | -.031 |
|  | JSS8 | .030 | -.019 | -.004 | -.016 | .007 | .034 |
|  | JSS9 | .098 | .128 | .046 | .046 | .049 | .016 |
|  | JSS10 | .070 | .050 | -.001 | -.009 | -.029 | -.013 |
|  | TIS1 | -.088 | -.069 | -.054 | -.019 | -.016 | -.023 |
|  | TIS2 | -.050 | -.047 | -.003 | -.012 | .003 | .009 |
|  | TIS3 | -.066 | -.039 | -.049 | -.026 | -.032 | .000 |
|  | TIS4 | -.103 | -.096 | -.031 | -.034 | -.025 | -.022 |
|  | TIS5 | -.012 | -.029 | .008 | .038 | .039 | .065 |
|  | TIS6 | -.077 | -.029 | -.037 | -.059 | -.043 | -.046 |

| **Correlation Matrix 2** | | | | | | | | |
| --- | --- | --- | --- | --- | --- | --- | --- | --- |
|  | | UWES7.Ded | UWES8.Abs | UWES9.Abs | EWWS1.Inter | EWWS2.Inter | EWWS3.Inter |  |
| Correlation | UWES1.Vig | .493 | .465 | .350 | .000 | -.048 | -.024 |  |
|  | UWES2.Vig | .538 | .521 | .343 | -.020 | -.039 | -.005 |  |
|  | UWES3.Ded | .650 | .596 | .350 | -.031 | -.045 | -.001 |  |
|  | UWES4.Ded | .651 | .603 | .405 | -.097 | -.106 | -.057 |  |
|  | UWES5.Vig | .576 | .526 | .347 | -.076 | -.059 | -.073 |  |
|  | UWES6.Abs | .490 | .408 | .304 | -.095 | -.101 | -.075 |  |
|  | UWES7.Ded | 1.000 | .639 | .377 | -.106 | -.099 | -.047 |  |
|  | UWES8.Abs | .639 | 1.000 | .634 | -.098 | -.109 | -.014 |  |
|  | UWES9.Abs | .377 | .634 | 1.000 | -.105 | -.085 | -.078 |  |
|  | EWWS1.Inter | -.106 | -.098 | -.105 | 1.000 | .730 | .681 |  |
|  | EWWS2.Inter | -.099 | -.109 | -.085 | .730 | 1.000 | .757 |  |
|  | EWWS3.Inter | -.047 | -.014 | -.078 | .681 | .757 | 1.000 |  |
|  | EWWS4.Inter | -.080 | -.058 | -.048 | .587 | .673 | .581 |  |
|  | EWWS5.Intra | -.140 | -.058 | .005 | .551 | .539 | .550 |  |
|  | EWWS6.Intra | -.046 | .010 | -.016 | .394 | .325 | .396 |  |
|  | EWWS7.Intra | -.008 | .018 | -.011 | .281 | .293 | .276 |  |
|  | EWWS8.Intra | -.061 | .004 | .028 | .336 | .275 | .347 |  |
|  | JSS1 | .043 | .035 | .002 | .402 | .311 | .344 |  |
|  | JSS2 | -.105 | -.098 | -.050 | .701 | .796 | .656 |  |
|  | JSS3 | -.083 | -.009 | -.028 | .517 | .442 | .507 |  |
|  | JSS4 | -.069 | -.005 | -.041 | .200 | .103 | .191 |  |
|  | JSS5 | -.031 | .012 | -.018 | .380 | .323 | .309 |  |
|  | JSS6 | .008 | .007 | .033 | .186 | .191 | .192 |  |
|  | JSS7 | -.097 | -.018 | .008 | .125 | .141 | .103 |  |
|  | JSS8 | .008 | -.041 | .004 | .246 | .226 | .215 |  |
|  | JSS9 | .012 | .075 | .048 | .256 | .232 | .268 |  |
|  | JSS10 | -.029 | .023 | .031 | .368 | .314 | .393 |  |
|  | TIS1 | -.009 | -.057 | -.013 | -.253 | -.222 | -.272 |  |
|  | TIS2 | .025 | -.014 | -.003 | -.282 | -.258 | -.309 |  |
|  | TIS3 | -.084 | -.061 | -.044 | -.278 | -.240 | -.300 |  |
|  | TIS4 | .006 | -.054 | -.004 | -.308 | -.224 | -.320 |  |
|  | TIS5 | .066 | .013 | .041 | -.332 | -.279 | -.353 |  |
|  | TIS6 | -.005 | -.021 | -.036 | -.249 | -.258 | -.296 |  |

| **Correlation Matrix 3** | | | | | | | |
| --- | --- | --- | --- | --- | --- | --- | --- |
|  | | EWWS4.Inter | EWWS5.Intra | EWWS6.Intra | EWWS7.Intra | EWWS8.Intra | JSS1 |
| Correlation | UWES1.Vig | .035 | .039 | .066 | .017 | .057 | .092 |
|  | UWES2.Vig | -.002 | .026 | .047 | .018 | .028 | .100 |
|  | UWES3.Ded | -.002 | -.028 | .019 | .001 | -.004 | .076 |
|  | UWES4.Ded | -.067 | -.046 | -.012 | .007 | -.034 | .032 |
|  | UWES5.Vig | -.018 | -.019 | -.053 | -.026 | -.004 | .020 |
|  | UWES6.Abs | -.060 | -.067 | -.032 | -.003 | -.003 | -.007 |
|  | UWES7.Ded | -.080 | -.140 | -.046 | -.008 | -.061 | .043 |
|  | UWES8.Abs | -.058 | -.058 | .010 | .018 | .004 | .035 |
|  | UWES9.Abs | -.048 | .005 | -.016 | -.011 | .028 | .002 |
|  | EWWS1.Inter | .587 | .551 | .394 | .281 | .336 | .402 |
|  | EWWS2.Inter | .673 | .539 | .325 | .293 | .275 | .311 |
|  | EWWS3.Inter | .581 | .550 | .396 | .276 | .347 | .344 |
|  | EWWS4.Inter | 1.000 | .480 | .294 | .240 | .212 | .155 |
|  | EWWS5.Intra | .480 | 1.000 | .524 | .391 | .504 | .400 |
|  | EWWS6.Intra | .294 | .524 | 1.000 | .546 | .533 | .338 |
|  | EWWS7.Intra | .240 | .391 | .546 | 1.000 | .415 | .220 |
|  | EWWS8.Intra | .212 | .504 | .533 | .415 | 1.000 | .460 |
|  | JSS1 | .155 | .400 | .338 | .220 | .460 | 1.000 |
|  | JSS2 | .679 | .567 | .348 | .307 | .295 | .358 |
|  | JSS3 | .290 | .510 | .558 | .366 | .543 | .472 |
|  | JSS4 | .090 | .199 | .266 | .162 | .265 | .246 |
|  | JSS5 | .241 | .358 | .294 | .239 | .380 | .436 |
|  | JSS6 | .204 | .376 | .219 | .235 | .334 | .226 |
|  | JSS7 | .075 | .183 | .260 | .179 | .253 | .213 |
|  | JSS8 | .137 | .320 | .426 | .340 | .514 | .350 |
|  | JSS9 | .172 | .277 | .242 | .261 | .340 | .341 |
|  | JSS10 | .231 | .516 | .588 | .444 | .613 | .481 |
|  | TIS1 | -.117 | -.378 | -.424 | -.289 | -.496 | -.425 |
|  | TIS2 | -.187 | -.409 | -.410 | -.350 | -.492 | -.342 |
|  | TIS | -.149 | -.328 | -.313 | -.129 | -.409 | -.416 |
|  | TIS4 | -.149 | -.397 | -.469 | -.326 | -.519 | -.406 |
|  | TIS5 | -.202 | -.395 | -.425 | -.302 | -.458 | -.386 |
|  | TIS6 | -.215 | -.413 | -.393 | -.352 | -.429 | -.297 |

| **Correlation Matrix 4** | | | | | | | | |
| --- | --- | --- | --- | --- | --- | --- | --- | --- |
|  | | JSS2 | JSS3 | JSS4 | JSS5 | JSS6 | JSS7 | JSS8 |
| Correlation | UWES1.Vig | -.019 | .023 | -.042 | .021 | .049 | .042 | .030 |
|  | UWES2.Vig | -.011 | .013 | -.058 | .015 | .030 | .014 | -.019 |
|  | UWES3.Ded | -.038 | .018 | -.015 | -.022 | .032 | -.006 | -.004 |
|  | UWES4.Ded | -.060 | -.028 | -.040 | -.059 | -.035 | -.036 | -.016 |
|  | UWES5.Vig | -.069 | -.046 | -.075 | -.025 | .022 | -.019 | .007 |
|  | UWES6.Abs | -.120 | -.085 | -.117 | -.083 | -.031 | -.031 | .034 |
|  | UWES7.Ded | -.105 | -.083 | -.069 | -.031 | .008 | -.097 | .008 |
|  | UWES8.Abs | -.098 | -.009 | -.005 | .012 | .007 | -.018 | -.041 |
|  | UWES9.Abs | -.050 | -.028 | -.041 | -.018 | .033 | .008 | .004 |
|  | EWWS1.Inter | .701 | .517 | .200 | .380 | .186 | .125 | .246 |
|  | EWWS2.Inter | .796 | .442 | .103 | .323 | .191 | .141 | .226 |
|  | EWWS3.Inter | .656 | .507 | .191 | .309 | .192 | .103 | .215 |
|  | EWWS4.Inter | .679 | .290 | .090 | .241 | .204 | .075 | .137 |
|  | EWWS5.Intra | .567 | .510 | .199 | .358 | .376 | .183 | .320 |
|  | EWWS6.Intra | .348 | .558 | .266 | .294 | .219 | .260 | .426 |
|  | EWWS7.Intra | .307 | .366 | .162 | .239 | .235 | .179 | .340 |
|  | EWWS8.Intra | .295 | .543 | .265 | .380 | .334 | .253 | .514 |
|  | JSS1 | .358 | .472 | .246 | .436 | .226 | .213 | .350 |
|  | JSS2 | 1.000 | .444 | .100 | .352 | .197 | .136 | .250 |
|  | JSS3 | .444 | 1.000 | .367 | .427 | .311 | .272 | .374 |
|  | JSS4 | .100 | .367 | 1.000 | .306 | .126 | .182 | .256 |
|  | JSS5 | .352 | .427 | .306 | 1.000 | .264 | .166 | .238 |
|  | JSS6 | .197 | .311 | .126 | .264 | 1.000 | .293 | .215 |
|  | JSS7 | .136 | .272 | .182 | .166 | .293 | 1.000 | .292 |
|  | JSS8 | .250 | .374 | .256 | .238 | .215 | .292 | 1.000 |
|  | JSS9 | .231 | .401 | .142 | .219 | .176 | .108 | .229 |
|  | JSS10 | .343 | .692 | .312 | .350 | .372 | .316 | .486 |
|  | TIS1 | -.236 | -.515 | -.300 | -.256 | -.289 | -.322 | -.366 |
|  | TIS2 | -.236 | -.433 | -.211 | -.299 | -.300 | -.255 | -.451 |
|  | TIS3 | -.234 | -.409 | -.306 | -.333 | -.240 | -.222 | -.382 |
|  | TIS4 | -.242 | -.495 | -.321 | -.301 | -.266 | -.307 | -.437 |
|  | TIS5 | -.258 | -.465 | -.338 | -.325 | -.190 | -.070 | -.333 |
|  | TIS6 | -.245 | -.384 | -.137 | -.234 | -.328 | -.220 | -.279 |

| **Correlation Matrix 5** | | | | | | | |
| --- | --- | --- | --- | --- | --- | --- | --- |
|  | | JSS9 | JSS10 | TIS21 | TIS2 | TIS3 | TIS4 |
| Correlation | UWES1.Vig | .098 | .070 | -.088 | -.050 | -.066 | -.103 |
|  | UWES2.Vig | .128 | .050 | -.069 | -.047 | -.039 | -.096 |
|  | UWES3.Ded | .046 | -.001 | -.054 | -.003 | -.049 | -.031 |
|  | UWES4.Ded | .046 | -.009 | -.019 | -.012 | -.026 | -.034 |
|  | UWES5.Vig | .049 | -.029 | -.016 | .003 | -.032 | -.025 |
|  | UWES6.Abs | .016 | -.013 | -.023 | .009 | .000 | -.022 |
|  | UWES7.Ded | .012 | -.029 | -.009 | .025 | -.084 | .006 |
|  | UWES8.Abs | .075 | .023 | -.057 | -.014 | -.061 | -.054 |
|  | UWES9.Abs | .048 | .031 | -.013 | -.003 | -.044 | -.004 |
|  | EWWS1.Inter | .256 | .368 | -.253 | -.282 | -.278 | -.308 |
|  | EWWS2.Inter | .232 | .314 | -.222 | -.258 | -.240 | -.224 |
|  | EWWS3.Inter | .268 | .393 | -.272 | -.309 | -.300 | -.320 |
|  | EWWS4.Inter | .172 | .231 | -.117 | -.187 | -.149 | -.149 |
|  | EWWS5.Intra | .277 | .516 | -.378 | -.409 | -.328 | -.397 |
|  | EWWS6.Intra | .242 | .588 | -.424 | -.410 | -.313 | -.469 |
|  | EWWS7.Intra | .261 | .444 | -.289 | -.350 | -.129 | -.326 |
|  | EWWS8.Intra | .340 | .613 | -.496 | -.492 | -.409 | -.519 |
|  | JSS1 | .341 | .481 | -.425 | -.342 | -.416 | -.406 |
|  | JSS2 | .231 | .343 | -.236 | -.236 | -.234 | -.242 |
|  | JSS3 | .401 | .692 | -.515 | -.433 | -.409 | -.495 |
|  | JSS4 | .142 | .312 | -.300 | -.211 | -.306 | -.321 |
|  | JSS5 | .219 | .350 | -.256 | -.299 | -.333 | -.301 |
|  | JSS6 | .176 | .372 | -.289 | -.300 | -.240 | -.266 |
|  | JSS7 | .108 | .316 | -.322 | -.255 | -.222 | -.307 |
|  | JSS8 | .229 | .486 | -.366 | -.451 | -.382 | -.437 |
|  | JSS9 | 1.000 | .409 | -.292 | -.268 | -.243 | -.267 |
|  | JSS10 | .409 | 1.000 | -.558 | -.535 | -.406 | -.561 |
|  | TIS1 | -.292 | -.558 | 1.000 | .472 | .467 | .709 |
|  | TIS2 | -.268 | -.535 | .472 | 1.000 | .388 | .561 |
|  | TIS3 | -.243 | -.406 | .467 | .388 | 1.000 | .539 |
|  | TIS4 | -.267 | -.561 | .709 | .561 | .539 | 1.000 |
|  | TIS5 | -.344 | -.507 | .515 | .428 | .410 | .572 |
|  | TIS6 | -.204 | -.496 | .403 | .527 | .284 | .445 |

| **Correlation Matrix 6** | | | |
| --- | --- | --- | --- |
|  | | TIS5 | TIS6 |
| Correlation | UWES1.Vig | -.012 | -.077 |
|  | UWES2.Vig | -.029 | -.029 |
|  | UWES3.Ded | .008 | -.037 |
|  | UWES4.Ded | .038 | -.059 |
|  | UWES5.Vig | .039 | -.043 |
|  | UWES6.Abs | .065 | -.046 |
|  | UWES7.Ded | .066 | -.005 |
|  | UWES8.Abs | .013 | -.021 |
|  | UWES9.Abs | .041 | -.036 |
|  | EWWS1.Inter | -.332 | -.249 |
|  | EWWS2.Inter | -.279 | -.258 |
|  | EWWS3.Inter | -.353 | -.296 |
|  | EWWS4.Inter | -.202 | -.215 |
|  | EWWS5.Intra | -.395 | -.413 |
|  | EWWS6.Intra | -.425 | -.393 |
|  | EWWS7.Intra | -.302 | -.352 |
|  | EWWS8.Intra | -.458 | -.429 |
|  | JSS1 | -.386 | -.297 |
|  | JSS2 | -.258 | -.245 |
|  | JSS3 | -.465 | -.384 |
|  | JSS4 | -.338 | -.137 |
|  | JSS5 | -.325 | -.234 |
|  | JSS6 | -.190 | -.328 |
|  | JSS7 | -.070 | -.220 |
|  | JSS8 | -.333 | -.279 |
|  | JSS9 | -.344 | -.204 |
|  | JSS10 | -.507 | -.496 |
|  | TIS1 | .515 | .403 |
|  | TIS2 | .428 | .527 |
|  | TIS3 | .410 | .284 |
|  | TIS4 | .572 | .445 |
|  | TIS5 | 1.000 | .318 |
|  | TIS6 | .318 | 1.000 |

**Key**:

| **Abbreviation** | **Full Name** |
| --- | --- |
| UWES | Utrecht Work Engagement Scale |
| Vig | Vigour |
| Ded | Dedication |
| Abs | Absorption |
| EWWS | Eudaimonic Workplace Wellbeing Scale |
| Inter | Interpersonal |
| Intra | Intrapersonal |
| JSS | Job Satisfaction Scale |
| TIS | Turnover Intention Scale |
