# Supplementary material for: Flourishing and job satisfaction in employees working in UK clinical trial units: a national cross-sectional survey
Source: BMC Health Serv Res. 2024 Dec 2;24:1522. doi: 10.1186/s12913-024-11986-x (PMC11610179; doi:10.1186/s12913-024-11986-x)
Supplement: Supplementary file 3 — Supplementary Material 3. [file 12913_2024_11986_MOESM3_ESM.docx]

**Supplementary Material 3: Interpretation of FACTS Scales**

**3.1 UWES Score Interpretation^1^.**

|  | **Vigour** | **Dedication** | **Absorption** |
| --- | --- | --- | --- |
| Very Low | ≤ 2.00 | ≤ 1.33 | ≤ 1.17 |
| Low | 2.01 – 3.25 | 1.34 – 2.90 | 1.18 – 2.33 |
| Average | 3.26 – 4.80 | 2.91 – 4.70 | 2.34 – 4.20 |
| High | 4.81 – 5.65 | 4.71 – 5.69 | 4.21 – 5.33 |
| Very High | ≥ 5.66 | ≥ 5.70 | ≥ 5.34 |

^1^ *^Schaufeli W, Bakker A. The Utrecht Work Engagement Scale (UWES) Manual. Occupational Health Psychology Unit, Utrecht University. 2004; p.1-58.^*

**3.2 JSS Score Interpretation^1^.**

|  | **Job Satisfaction** |
| --- | --- |
| Very Low | 10-26 |
| Low | 27-31 |
| Average | 32-38 |
| High | 39-41 |
| Very High | 42-50 |

*^1 Macdonald S, Maclntyre P. The generic job satisfaction scale: Scale development and its correlates. Employee Assistance Quarterly. 1997;13(2):p.1-16.^*

**3.3 Eudaimonic Workplace Wellbeing Scale (EWWS) (flourishing).**

The EWWS does not have a formal scoring manual or interpretation/classification criteria. As such, we performed a literature search to identify peer-reviewed published research which used the EWWS and reported mean scores for their sample. The table below shows Mean and Standard Deviation scores for our sample (in the main manuscript we report Mean and Standard Error Means) so that we could make appropriate comparisons. We also report these descriptive statistics based on total raw score and average scores, since the papers identified used both of these approaches and there is no clear guidance on best practice for this scale.

|  | **Total Raw Scores (Mean±SD)** | | **Average Score (Mean±SD)** | | |
| --- | --- | --- | --- | --- | --- |
| *Flourishing* | *UK CTU staff (n=484)* | *Saudi employees (n=395)^1^* | *UK CTU staff (n=484)* | *Employees, location not stated (n=1346)^2^* | *Dutch employees (n=435)^3^* |
| **Interpersonal** | 14.22±3.27 | 16.89±2.86 | 3.55±.81 | 3.70 ±0.67 | 3.89±.65 |
| **Intrapersonal** | 14.77±2.89 | 17.92±2.11 | 3.69±.72 | 3.77±.63 | 3.98± .63 |

*^1 Al Kahtani, N. S., & MM, S. (2022). A study on how psychological capital, social capital, workplace wellbeing, and employee engagement relate to task performance. Sage Open, 12(2), 21582440221095010.^*

*^2 Bartels, A. L., Peterson, S. J., & Reina, C. S. (2019). Understanding well-being at work: Development and validation of the eudaimonic workplace well-being scale. PloS one, 14(4), e0215957.^*

*^3 Vries, A. D., Broks, V. M., Bloemers, W., Kuntze, J., & De Vries, R. E. (2022). Self-, other-, and meta-perceptions of personality: Relations with burnout symptoms and eudaimonic workplace well-being. Plos one, 17(7), e0272095.^*

**3.4 Turnover Intention Scale (TIS)**

The TIS does not have a formal scoring manual. As such, we performed a literature search to identify peer-reviewed published research which used the TIS and reported mean scores for their sample. The table below shows the Mean and Standard Deviation scores for our sample (in the main manuscript we report Mean and Standard Error Means) so that we could make appropriate comparisons.

|  | **Total Mean Score (Mean±SD)** | | |
| --- | --- | --- | --- |
|  | UK CTU staff (n=484) | Hungarian-based staff (n=269)^1^ | South African-based staff (n=79)^2^ |
| **Turnover Intention** | 2.75±1.06 | 2.46±1.26 | 2.86±1.08 |

*^1 Oosthuizen, R. M., Coetzee, M., & Munro, Z. (2016). Work-life balance, job satisfaction and turnover intention amongst information technology employees. Southern African Business Review, 20, 446-467^*

*^2 Németh, Z., Deák, P., Szűcs, R. et al. (2024) Validation of the Hungarian version of the 6-item turnover intention scale among elderly care workers. Sci Rep 14, 15593. https://doi.org/10.1038/s41598-024-66671-0^*
